# UTILIZING THE PMDC AS A FRAMEWORK FOR THE PROVISION OF PERSON-CENTERED CARE IN NURSING HOME SETTINGS

**DOI:** 10.1093/geroni/igae098.1096

**Published:** 2024-12-31

**Authors:** Bill Buron, Kathy Wright

**Affiliations:** Chair; Discussant

## Abstract

This symposium showcases the research of the Geropsychiatric Nursing Collaborative, a workgroup affiliated with the NHCGNE. Kitwood (1997) defined Personhood, as ‘a standing or status that is bestowed on one human being, by another in the context of relationship and social being.’ Buron (2008) designed the Personhood Model for Dementia Care (PMDC) based on Kitwood’s definition and Maslow’s (1943) Hierarchy of Needs Theory. The PMDC is an intuitive framework to promote the identification and utilization of person-centered care (PCC) interventions in nursing homes. This symposium begins with an overview of the model as a framework for an integrative review of effective PCC-focused interventions. Next, three specific interventions will be discussed and highlighted, each representing at least one level of personhood as defined and described in the PMDC. The first illustrates how biological needs for nutrition intersect with relational aspects of care during mealtime and introduces an effective strategy for assisting RLWD with their meals. The researchers found more PCC and less task-centered care were associated with improved mealtime behaviors and/or food intake in RLWD. In the next example, the researchers highlight individual personhood through an exploration of resident preferences. The researchers found residents have diverse approaches to expressing their preferences and utilize a range of resident-led, family-involved, and staff-brokered strategies to meet preferences. In the final example, researchers developed an on-line person-centered dementia training for undergraduate nursing students. The aim of this training was to improve nursing care of people living with dementia and highlights the importance of sociologic personhood. Nursing Care for Older Adults Interest Group Sponsored Symposium

